# Spontaneous bilateral extrapleural hematoma: a case report

**DOI:** 10.1186/s13019-015-0300-3

**Published:** 2015-07-14

**Authors:** Sheng-I Hu, Shih-Chun Lee, Hung Chang, Yen-Shou Kuo

**Affiliations:** 1 Division of Colon & Rectal Surgery, Tri-Service General Hospital, National Defense Medical Center, 325, Cheng-Kung Road 2nd Section, Taipei, 114 Taiwan; 2 Division of Thoracic Surgery, Tri-Service General Hospital, National Defense Medical Center, 325, Cheng-Kung Road 2nd Section, Taipei, 114 Taiwan

**Keywords:** Extrapleural hematoma, hemodialysis, AV fistula

## Abstract

Extrapleural hematoma (EPH) is a rare condition characterized by the accumulation of blood in the extrapleural space. EPH is generally identified by computed tomography (CT), which shows an inward displacement of extrapleural fat due to intrathoracic peripheral fluid accumulation (Ann Ital Chir 75(83): 5, 2004; J Korean Radiol Soc 49: 89–97, 2003; Monaldi Arch Chest Dis 63(3): 166–169, 2005). EPH has been reported to be associated with chest trauma and injuries. However, the correlation between hemodialysis and EPH has not yet been reported. The causes of EPH in a hemodialysis patient have been postulated, which include high venous flow through the arteriovenous fistula that results in an increase in venous pressure stenosis and/or thrombosis of the brachiocephalic and/or subclavian veins. These conditions thereby induce an increase in venous pressure in the intercostals and bronchial veins of the chest. Pleural fluid resorption is rare and excess pleural fluid formation commonly occurs (J Thoracic Imaging 26(3): 218–223, 2011). The occurrence of pleuritis with fusion of the two pleuric layers results in hematoma development in the extrapleural space instead of the pleural space. We present a chronic hemodialysis patient with spontaneous unilateral EPH. The progression to bilateral EPH was noted after VATS procedure. Awareness of EPH and the use of conservative management are key points for the treatment of this rare clinical condition.

## Background

Spontaneous extrapleural hematoma (EPH) is a rare disorder. We present a case of a chronic hemodialysis patient with spontaneous EPH. Surgical intervention was performed but was unsucessful, and the patient eventually died. In this report, we review the literature on spontaneous EPH as well as discuss the etiology and possible solutions for this condition.

**Fig. 1:**
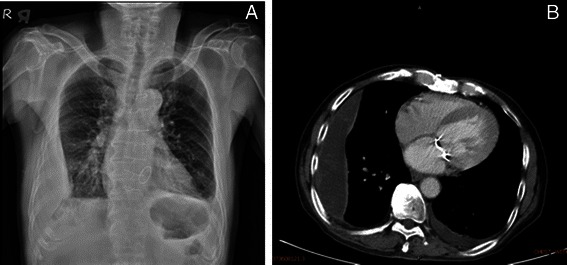
**a** Chest plain film generated at our emergency department showing the “fat sign” in the right lower thorax. **b** Chest CT showing a unilateral (right side) extrapleural hematoma

**Fig. 2:**
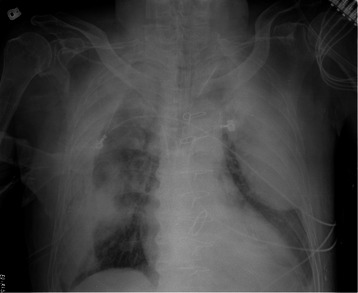
Post-operative chest plain film showing the progression of the right extrapleural hematoma. A newly developed left extrapleural hematoma was noted

## Case presentation

A 66-year-old male was admitted to our facility with a chief complaint of low-grade fever (body temperature: 37.8 °C) and progressive exertional dyspnea for past 3 days. The patient underwent regular hemodialysis for 10 years as treatment for diabetic nephropathy. He had a history of valvular heart disease with severe mitral regurgitation with congestive heart failure and underwent mitral valve repair 2 years prior to consultation. Regular outpatient department follow-up with oral ticlopidine at a dose of 100 mg daily was recorded. Neither recent medical history with surgical procedure nor recent trauma history was reported. The patient went to our emergency department and his chest plain film revealed unilateral (right side) fat-shaped pleural effusion (Fig. 1a). Chest CT imaging disclosed a right side EPH (Fig. 1b). Hemoglobin and hematocrit values were 7.5 g/dL and 29 %, respectively. Moreover, his white cell count and platelet count were 7,620/mm^3^ and 173,000/mm^3^, respectively. Prothrombin and partial thromboplastin times were determined to be 11 s and 31 s, respectively. His INR level was 1.1 s (0.8 s–1.2 s). Serum values for urea nitrogen and creatinine were 42 mg/dL and 5.7 mg/dL, respectively. A chest tube was placed in the right pleural area and 500 mL of hemorrhagic fluid was drained. Thoracoscopic surgery with evacuation of clots was performed on the first day after admission. Severe adhesion between visceral pleural and parietal pleura of the right lower lobe and old blood clots retained in the costophrenic angle and major fissure were noted. No active bleedings were observed. However, a progressive bilateral EPH developed after the operation (Fig. 2). Poor general condition with severe coagulopathy subsequently developed. Profound hypotension was also noted. A high-dose inotropic agent was administered and massive blood transfusion was performed; however, his condition continued to deteriorate. Finally, the patient expired on the fifth day after admission.

### Discussion

EPH most commonly occurs in high-energy blunt trauma, especially those involving rib fractures [1]. Diagnosis of EPH is generally difficult and often delayed and is mainly based on x-ray findings; therefore, a thoracic CT scan is usually conducted to generate a diagnosis at the shortest possible time. A typical CT finding in EPH is the “fat sign,” which is the displacement of the thoracic soft-tissue band medially to the ribs due to a fluid accumulation in the extrapleural space [2]. A stable patient and a small hematoma can be managed conservatively. Evacuation of blood clots is required in large EPHs because respiratory and circulatory disturbances may occur [3].

Hemothorax may also occur in EPH cases due to coagulopathy during hemodialysis, suggesting that it arises secondary to platelet dysfunction of uremia and/or anticoagulant use. Nevertheless, the incidence of unilateral EPH is relatively rare in this situation [4]. In the present report, the patient underwent anticoagulant medication for two years with an INR level of 1.1 s, suggesting that our EPH case was not associated with anticoagulant usage.

Salim et al. previously described a left side unilateral hemothorax in a 46-year-old South Indian male due to a giant arteriovenous (AV) hemodialysis fistula in the left forearm. The patient had enlarged artificial AV fistula without evidence of thrombosis or stenosis. Intercostal drainage was performed. After ligation of AV fistula, pleural effusion decreased and the patient was discharged after three days [4]. The high venous flow through the AV fistula caused an increase in venous pressure stenosis and/or thrombosis of the brachiocephalic and/or subclavian veins. Stenosis of the brachiocephalic vein in association with high venous flow rates can increase venous pressure in the intercostals and bronchial veins of the chest, which in turn could alter local hemodynamics. As pleural fluid resorption is poor in normal conditions, excess pleural fluid formation is expected to occur, leading to unilateral pleural effusion on the same side as that of vein stenosis/thrombosis [5]. Therefore, the hematoma collected in the extrapleural space instead of the pleural one is putative because of a previous pleuritis with fusion of the two pleuric layers. The possibility of unilateral spontaneous EPH due to high venous flow through the AV fistula should therefore be considered in this particular situation. In addition, ligation of the AV fistula should also be considered to control hemothorax.

Surgical intervention with tube thoracostomy or video-assisted thoracic surgery is an ineffective therapeutic option for EPH. In our present case, the progression of bilateral EPH was noted after surgery and aggressive conservative treatment was suggested. This particular situation is also strongly associated with a high mortality rate. Therefore, in this specific setting, ligation of the AV fistula was considered as an appropriate option. Nevertheless, a large series of cohort studies or randomized trials are necessary to establish its effectiveness and safety. Awareness of EPH and the use of conservative management are key points for the treatment of this rare clinical condition.

## Conclusions

Spontaneous EPH is a rare and life-threatening condition, and its treatment in a dialysis patient is particularly challenging. Awareness of EPH and the application of conservative management are critical points to consider in the treatment of this rare clinical condition.

## Consent

Written informed consent was obtained from the patient for publication of this Case report and any accompanying.
